# Topical arginase inhibition decreases growth of cutaneous squamous cell carcinoma

**DOI:** 10.1038/s41598-021-90200-y

**Published:** 2021-05-24

**Authors:** Amit Mittal, Mike Wang, Aurobind Vidyarthi, Diana Yanez, Gabriela Pizzurro, Durga Thakral, Erin Tracy, Oscar R. Colegio

**Affiliations:** 1 Department of Dermatology, Yale School of Medicine, New Haven, USA; 2 Department of Medicine, Massachusetts General Hospital, 55 Fruit Street, Boston, MA 02114 USA; 3 Department of Immunology, Roswell Park Comprehensive Cancer Center, Buffalo, USA; 4 Department of Dermatology, Roswell Park Comprehensive Cancer Center, Elm and Carlton Streets, Buffalo, NY 14263 USA

**Keywords:** Cancer immunotherapy, Squamous cell carcinoma

## Abstract

Cutaneous squamous cell carcinomas (cSCC) are among the most commonly diagnosed malignancies, causing significant morbidity and mortality. Tumor-associated macrophage (TAM) expression of arginase is implicated in tumor progression, and therapeutic use of arginase inhibitors has been studied in various cancers. However, investigating potential cSCC immunotherapies including arginase inhibition in pre-clinical models is hampered by the lack of appropriate tumor models in immunocompetent mice. PDV is a cSCC cell line derived from chemical carcinogenesis of mouse keratinocytes. PDVC57 cells were derived from a PDV tumor in C57BL/6 (B6) mice. Unlike PDV, PDVC57 tumors grow consistently in B6 mice, and have increased TAMs, decreased dendritic and T cell intra-tumor infiltration. Arginase inhibition in cSCC tumors using Nω-hydroxy-nor-arginine (nor-NOHA) reduced tumor growth in B6 mice but not immunodeficient *Rag1*-deficient mice. nor-NOHA administration increased dendritic and T cell tumor-infiltration and PD-1 expression. The combination of nor-NOHA and anti-PD-1 therapy with nivolumab enhanced anti-PD-1 therapeutic efficacy. This study demonstrates the therapeutic potential of transcutaneous arginase inhibition in cSCC. A competent immune microenvironment is required for tumor growth inhibition using this arginase inhibitor. Synergistic co-inhibition of tumor growth in these results, supports further examination of transcutaneous arginase inhibition as a therapeutic modality for cSCC.

## Introduction

Cutaneous squamous cell carcinoma (cSCC) is the second most common cancer that develops in humans in the United States of America^1,2^. It is estimated that over 700,000 new cases of cSCC are diagnosed annually in the United States^3^. Whereas most cSCCs can be cured surgically, many cSCCs metastasize to lymph nodes and distant organs, causing significant morbidity and mortality. In 2012, up to 419,543 Caucasians were diagnosed with cSCC, up to 12,572 patients developed nodal metastases, and up to 8791 died from cSCC in the United States^4^.

Published data and clinical experience suggest that innate and adaptive immunity play crucial roles in the development, progression, and prognosis of cSCC. Solid organ transplant recipients (SOTR) require lifelong systemic immunosuppression to prevent graft rejection, and compared with the general population, SOTRs are approximately 100-times more likely to develop cSCC^5^. The cSCC in SOTR are generally more aggressive, being more invasive and metastatic^6,7^. Within the tumor microenvironment, cSCC demonstrate higher macrophage and T cell infiltration compared to normal skin or non-malignant keratinocyte-derived lesions such as actinic keratoses or keratoacanthomas^8,9^.

Tumor-associated macrophages (TAMs) are associated with poor prognosis in various cancers, including lung, colorectal, liver, and breast cancers^10–15^. TAMs facilitate tumor progression, proliferation and metastasis by stimulating angiogenesis and inhibiting antitumor T cell responses^16,17^. TAM expression of the enzyme arginase, which catalyzes hydrolysis of l-arginine to produce urea and l-ornithine and depletes extracellular l-arginine, has been implicated in tumor progression^18–20^. However, the mechanisms by which arginase are critical to tumor growth have yet to be fully elucidated.

While arginase inhibition provides a potentially useful tool in treating cSCC, investigating the cSCC tumor microenvironment in preclinical models presents a major challenge, as there is a lack of implantable syngeneic cSCC tumor models in immunocompetent mice. Implantable tumor models have been used extensively in T cell-deficient mice at the expense of accurately modeling innate and adaptive immune interactions.

In this study, we used PDVC57, an cSCC cell line derived from the C57BL/6-derived PDV cSCC cell line via in vivo passage, to serve as a murine tumor model for analyzing the immune microenvironment in cSCC^21,22^. In contrast to implantation of PDV cells, which develop tumors in ~ 10% of C57BL/6 mice, PDVC57 cells develop tumors in 100% of C57BL/6 mice. We investigated the role of arginase in PDVC57 tumor progression in vivo using transcutaneous inhibition of arginase. Inhibition of arginase activity was effective in reducing tumor growth in vivo. We determined that PD-1, a T cell activation/exhaustion marker and the prototypical checkpoint inhibitor target, was upregulated in the setting of small molecule inhibition of arginase. The combination of transcutaneous arginase inhibition with anti-PD-1 therapy demonstrated therapeutic synergy in the reduction of PDVC57 cSCC tumor growth.

## Results

### PDV and PDVC57 are genetically related murine squamous carcinoma cell lines that grow similarly in vitro but are distinct in their growth in vivo

**Figure 1:**
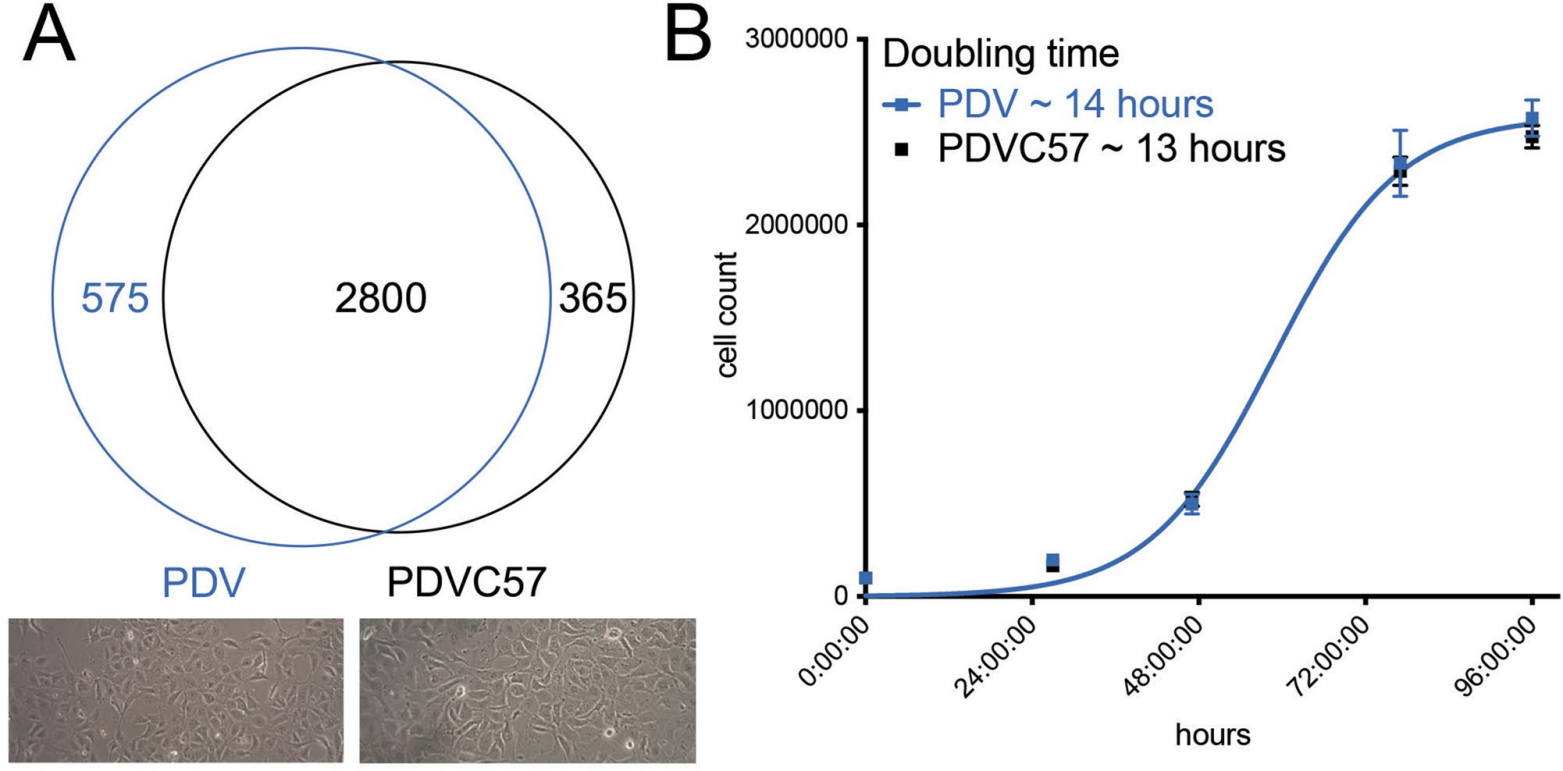
PDV and PDVC57 have similar exonic variants and doubling times in vitro. (**A**) Upper: whole exome sequencing revealed that the two cell lines shared 2800/3640 (76.9%) of exonic variants compared to normal mouse peripheral blood leukocytes; lower panels demonstrate light microscopy images of the cell lines growing in vitro, with PDVC57 (right) demonstrating greater size and granularity than PDV (left). (**B**) Logistic growth curves of the two cell lines that show no significant difference between the growth curves using the F-test, *P* = 0.6350.

To better characterize the PDVC57 cell line, PDV and PDVC57 cells were cultured and whole exome sequencing was performed. Sequencing demonstrated that PDV and PDVC57 share a majority (2800/3640, 76.9%) of their exonic variants compared to normal blood leukocytes (Fig. 1A, upper) and the two cell lines have similar mutational burdens (PDV: 2896 exonic mutations; PDVC57: 2726 exonic mutations. Supplemental Fig. 1). The cultured cells appeared morphologically different (Fig. 1A, lower) and flow cytometric analysis revealed that PDVC57 cells were significantly larger and more granular than PDV cells (Supplemental Fig. 2). Finally, the cell lines had similar logistic growth curves that demonstrated no significant difference between the two, with PDVC57 having a slightly shorter but not significantly different calculated doubling time (Fig. 1B).

**Figure 2:**
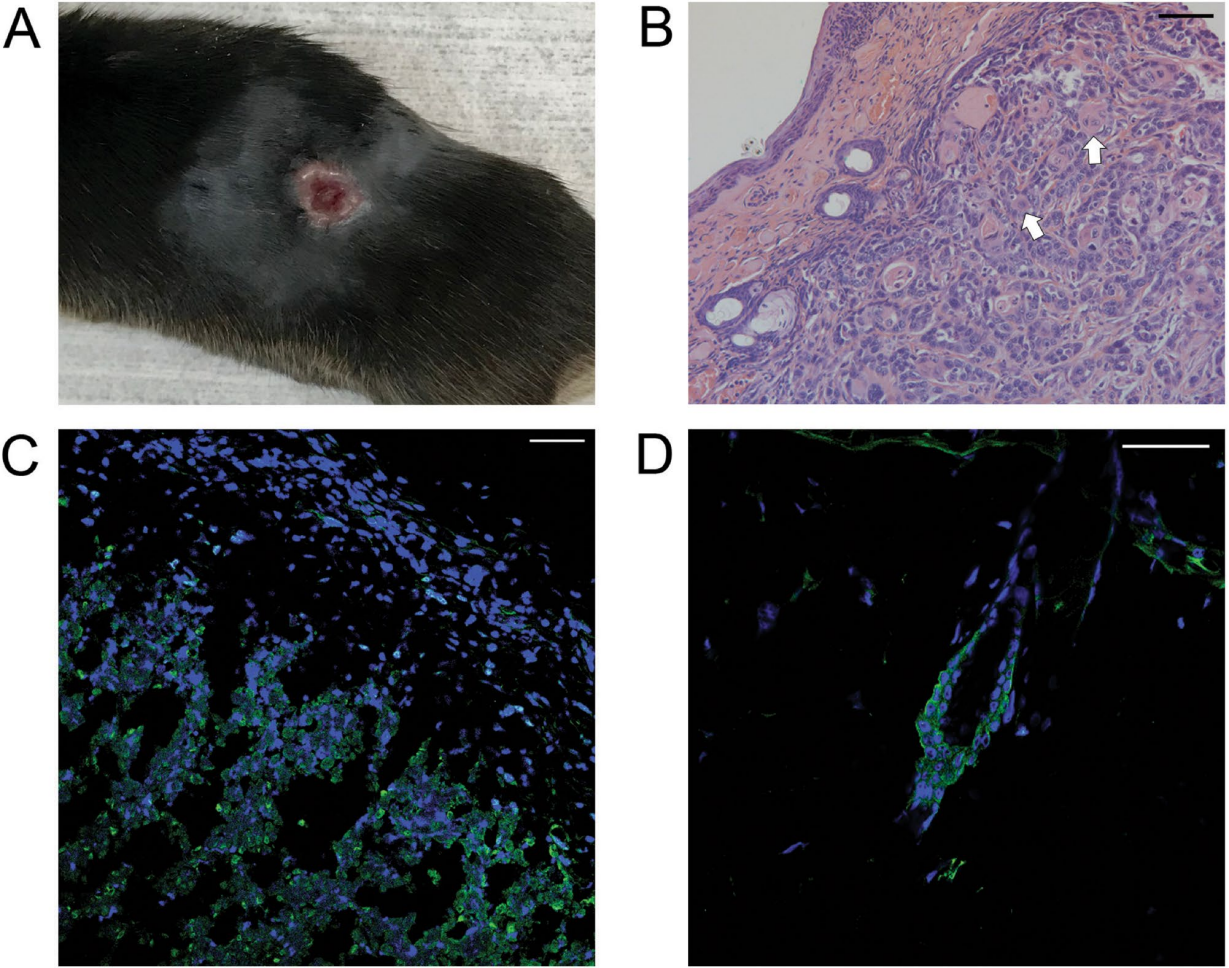
PDVC57 cells produce tumors consistent with cutaneous squamous cell carcinomas. (**A**) Injected tumor at day 21 demonstrated ulceration consistent with squamous cell carcinoma. (**B**) The histology of the injected tumors demonstrated cords and nests of malignant epithelial cells with nests of keratinizing cells in the dermal layer consistent with cutaneous squamous differentiation (white arrows). (**C**) The tumor with abnormal broad expression p63 (green) under immunofluorescence microscopic imaging, a marker of squamous differentiation; DAPI (blue) used to demonstrate cell nuclei. (**D**) The hair follicle with normal pattern of expression of p63 (green); DAPI (blue) used to demonstrate cell nuclei. Scale bars = 40 µm.

To develop and characterize this syngeneic cSCC tumor model, we injected 1, 5, or 10 million PDVC57 cells intradermally into the flanks of five C57BL/6 (B6) mice per group. All five mice in the 5 and 10 million cell injections developed tumors, and 3/5 (60%) of the mice in 1 million-cell injection developed tumors (Supplemental Fig. 3). The resultant tumors were harvested at day 21 (Fig. 2A) and either embedded in paraffin for histopathological analysis or frozen for immunofluorescence. The histology of the injected tumors demonstrated cords and nests of malignant epithelial cells with nests of keratinizing cells consistent with cutaneous squamous differentiation^23^ (Fig. 2B). Under immunofluorescence microscopic imaging, the tumors broadly expressed p63, a marker of squamous differentiation often used in the identification of SCCs and usually only found in the basal layer of the epidermis and the cutaneous appendages, such as the hair follicles^24–26^ (Fig. 2C,D).

**Figure 3:**
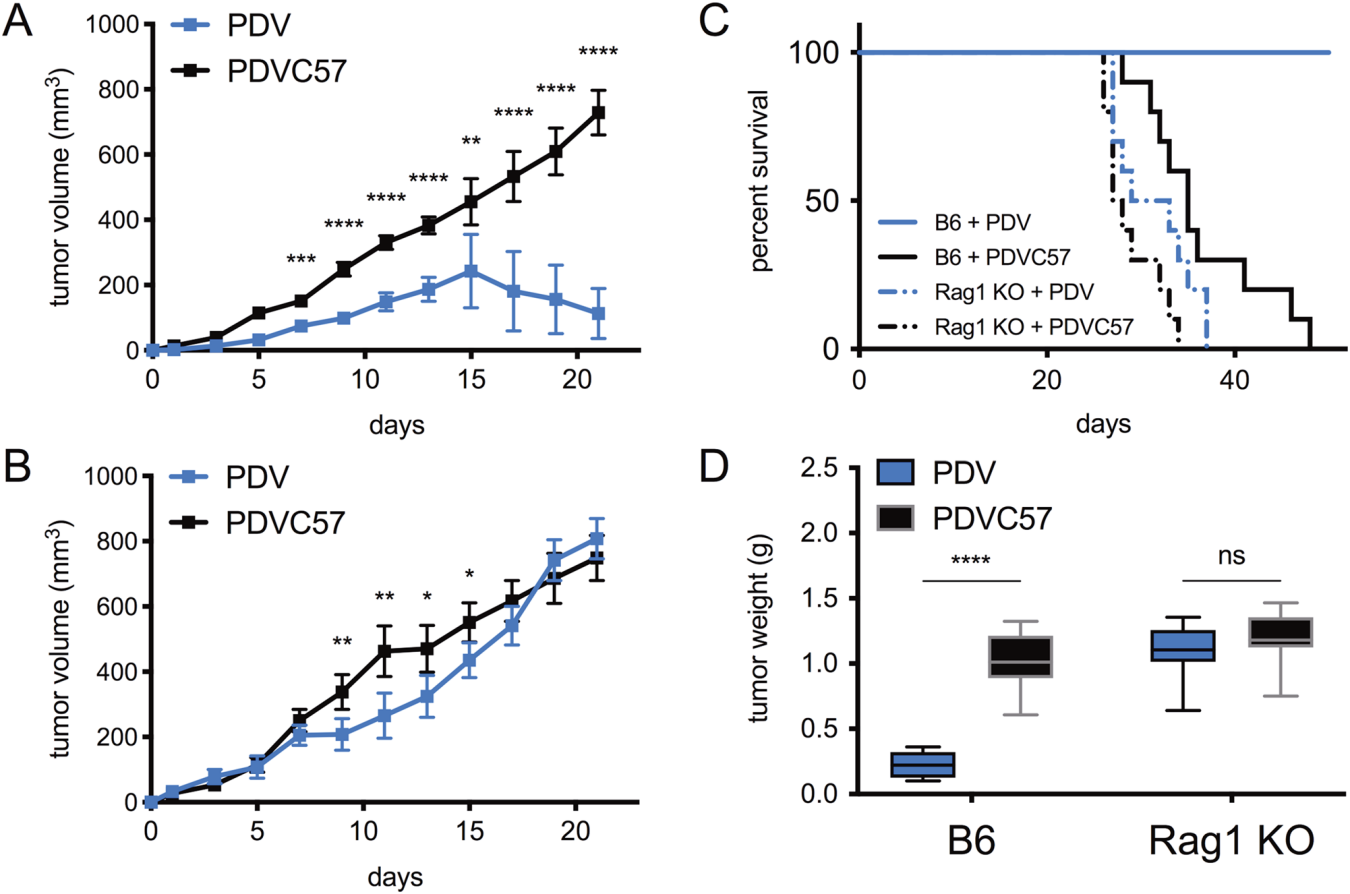
PDVC57 tumors grow in immunocompetent mice, unlike PDV tumors. (**A**) PDV tumors regress at approximately two weeks in immunocompetent B6 mice while PDVC57 continue to grow. (**B**) Both PDV and PDVC57 tumors continue to grow without regression in immunodeficient *Rag1* KO mice. (**C**) Kaplan–Meier survival curves of PDV and PDVC57 in either B6 or *Rag1* KO mice. Mantel-Cox test demonstrated significantly different survival curves, *P* < 0.0001. Post-hoc analysis demonstrates that in B6 mice, there exists a significant difference between PDV and PDVC57 tumors (*P* < 0.0001), while in Rag1 KO mice, there is no significant difference between PDV and PDVC57 tumors. (**D**) Endpoint tumor weights of PDV and PDVC57 in either B6 or *Rag1* KO mice. *P < 0.05; **P < 0.01; ***P < 0.001; ****P < 0.0001 in (**A**), (**B**), and (**D**) using the unpaired *t*-test adjusted for multiple comparisons. Two separate experiments with n > 10 per group.

Given that the PDVC57 cells consistently developed tumors in B6 mice with 5-million-cell injections, we intradermally injected two sets of 10 mice with 5 million PDV *versus* PDVC57 cells. Comparing PDV and PDVC57 intradermal tumors in B6 mice, injected PDV tumors grew more slowly, reached a growth peak at approximately day 15, and subsequently regressed (Fig. 3A). None of the ten B6 mice bearing PDV tumors reached the experimental endpoint and were all censored at day 42, and the tumor weights at the conclusion of the experiment were significantly lower than PDVC57 tumors (Fig. 3C,D). These differences were not demonstrated when the cells are injected into *Rag1*-deficient mice (B6.129S7-*Rag1*^*tm1Mom*^/J) mice, which lack mature T and B cells (Fig. 3B–D).

### Transcutaneous arginase inhibition is sufficient to inhibit PDVC57 tumor growth

**Figure 4:**
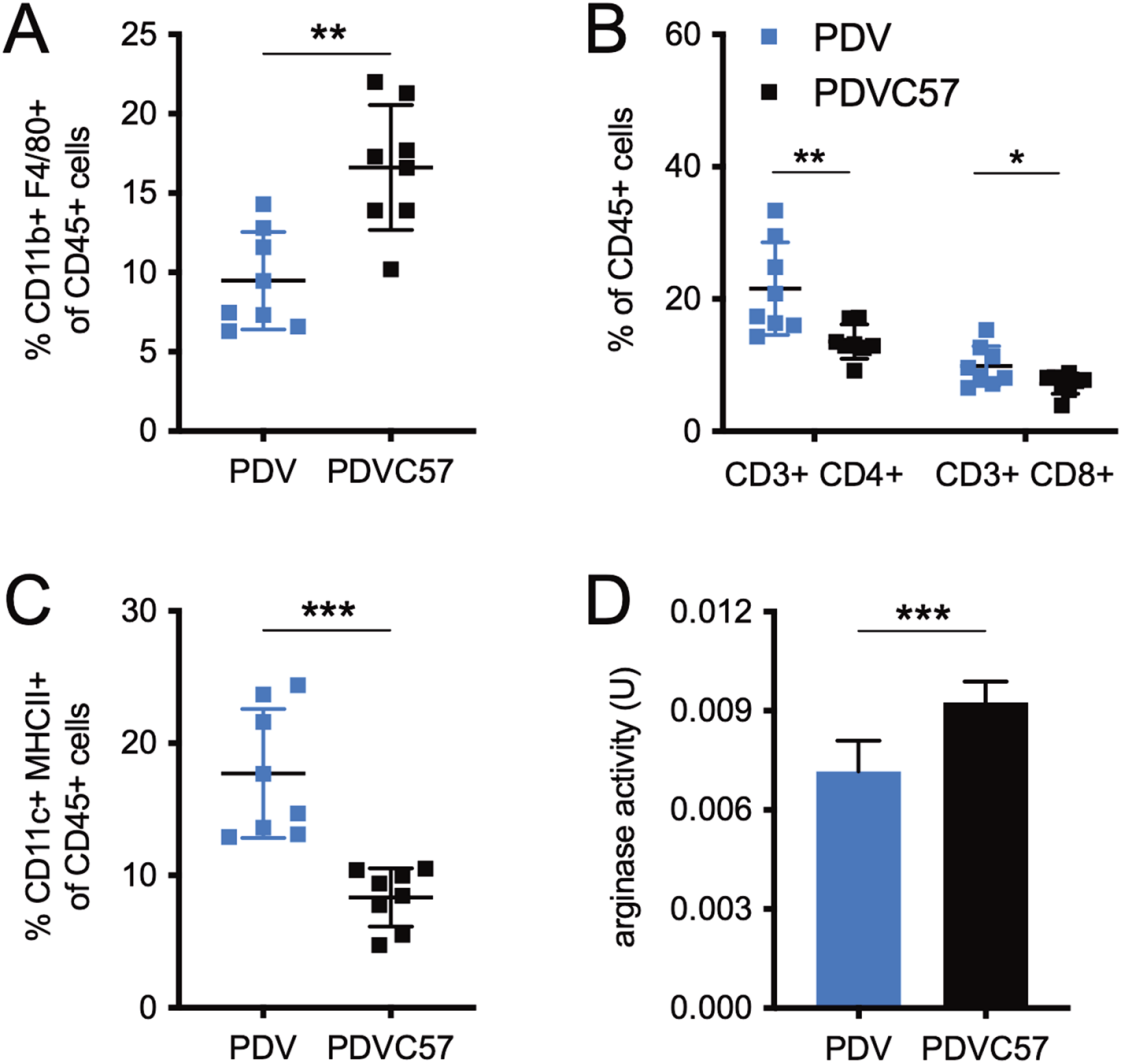
PDVC57 tumors recruit more tumor-associated macrophages and fewer T cells and dendritic cells than PDV tumors. (**A**) Percentage of intratumoral CD45^+^ leukocytes that are CD11b^+^ F4/80^+^, indicating tumor-associated macrophages; significantly higher in PDVC57 tumors. (**B**) Percentage of intratumoral CD45 + leukocytes that are CD3^+^ CD4^+^ or CD3^+^ CD8^+^, indicating tumor-infiltrating T cells; CD3^+^ CD4^+^, CD3^+^ CD8^+^ cells significantly lower in PDVC57 tumors. (**C**) Percentage of intratumoral CD45^+^ leukocytes that are CD11c^+^ MHCII^+^, indicating tumor-infiltrating dendritic cells; significantly lower in PDVC57 tumors. (**D**) Arginase activity in units of catalytic activity (U) as assessed by enzymatic fluorometric assay; significantly higher in PDVC57 tumors. *P < 0.05; **P < 0.01; ***P < 0.001 using the unpaired *t*-test adjusted for multiple comparisons. Two separate experiments with n > 5 per group.

The growth of PDV tumors in immunodeficient *Rag1* knockout (KO) mice (B6.129S7-*Rag1*^*tm1Mom*^/J) in contrast to the immunocompetent B6 mice demonstrates that PDV tumors are immunologically rejected in B6 mice; furthermore, PDVC57 tumors may modulate the immune microenvironment to promote tumor growth^27^. Flow cytometry of PDV and PDVC57 tumors injected into B6 mice showed that PDVC57 tumors contained more tumor-associated macrophages (TAM), fewer tumor-infiltrating dendritic cells, and fewer tumor-infiltrating CD4^+^ and CD8^+^ T cells (Fig. 4A–C). The increase in TAMs in SCC has been shown to lead to a worse prognosis, and previous work and existing literature had shown that TAM-derived arginase is important in promoting tumor growth^20,28,29^. In agreement, we observed that PDVC57 tumors demonstrated greater arginase activity compared to PDV tumors (Fig. 4D).

**Figure 5:**
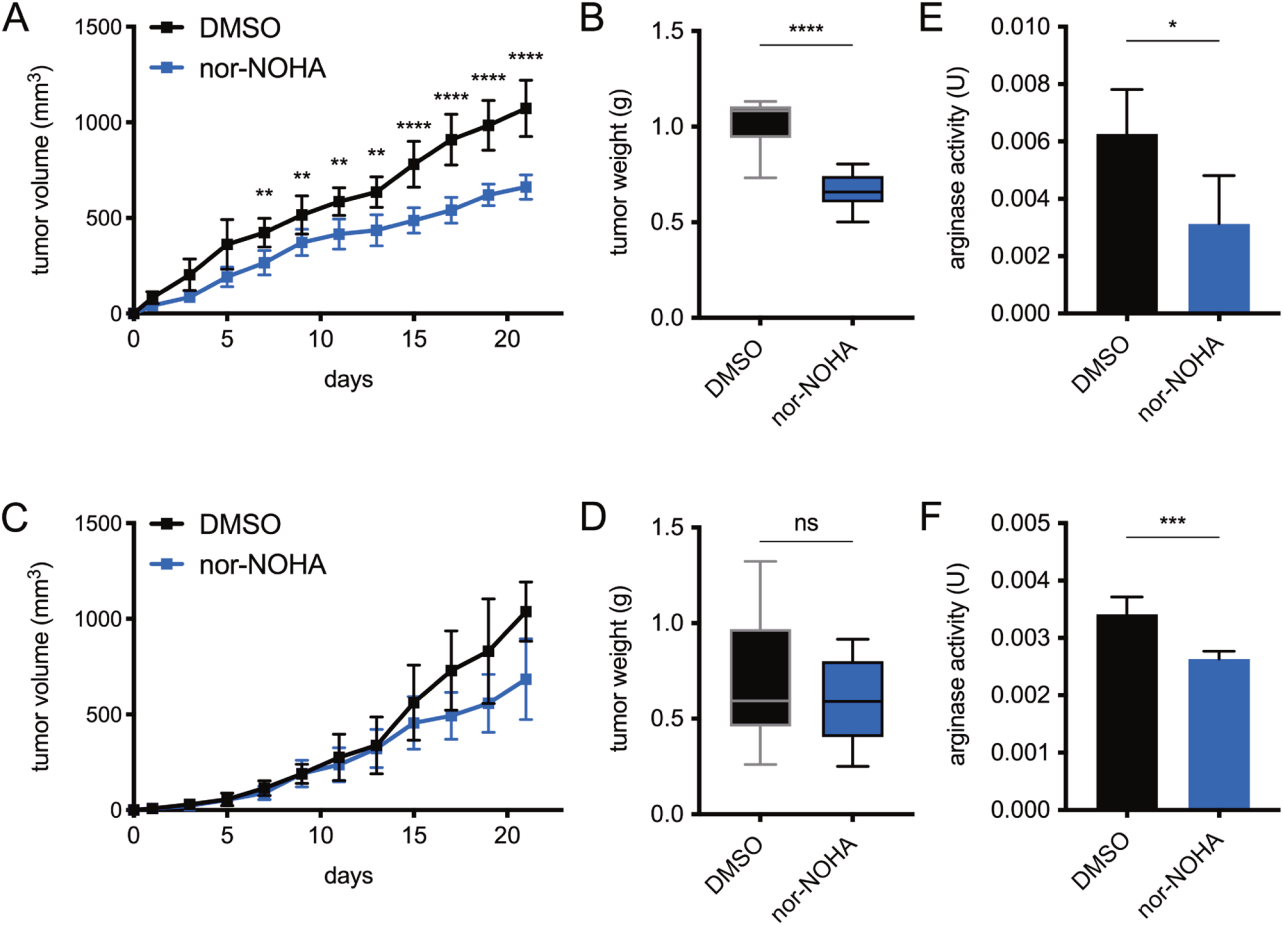
Transcutaneous arginase inhibition significantly reduces growth of PDVC57 tumors in immunocompetent but not immunodeficient mice. (**A**) Growth curves of treated PDVC57 tumors in immunocompetent B6 mice, with a significant difference by the end of week one. (**B**) Endpoint tumor weights of treated PDVC57 tumors in immunocompetent B6 mice, with a significant difference. (**C**) Growth curves of treated PDVC57 tumors in immunodeficient *Rag1* KO mice, with no significant differences. (**D**) Endpoint tumor weights of treated PDVC57 tumors in immunodeficient *Rag1* KO mice, with no significant difference. (**E**) Arginase activity as assessed by enzymatic fluorometric assay of treated PDVC57 tumors in immunocompetent B6 mice, which was significantly different indicating effective inhibition of arginase. (**F**) Arginase activity in units of catalytic activity (U) as assessed by enzymatic fluorometric assay of treated PDVC57 tumors in immunodeficient *Rag1* KO mice, which was significantly different indicating effective inhibition of arginase. *P < 0.05; **P < 0.01; ***P < 0.001; ****P < 0.0001 using the unpaired *t*-test adjusted for multiple comparisons. Three separate experiments with n > 5 per group.

Using a novel transcutaneous delivery model of arginase inhibition, Nω-hydroxy-nor-arginine (nor-NOHA) was topically applied to the cSCCs formed after intradermal PDVC57 injection. Compared to vehicle control, daily application of 5 mM nor-NOHA significantly decreased the volume and weight of PDVC57 tumors in WT B6 mice (Fig. 5A,B). This difference was not demonstrated in immunodeficient *Rag1* KO mice (Fig. 5C,D), while arginase activity assays confirmed that nor-NOHA was able to successfully decrease arginase activity in both experimental groups (Fig. 5E,F).

### Transcutaneous arginase inhibition and systemic PD-1 inhibition are synergistic in decreasing PDVC57 tumor growth

**Figure 6:**
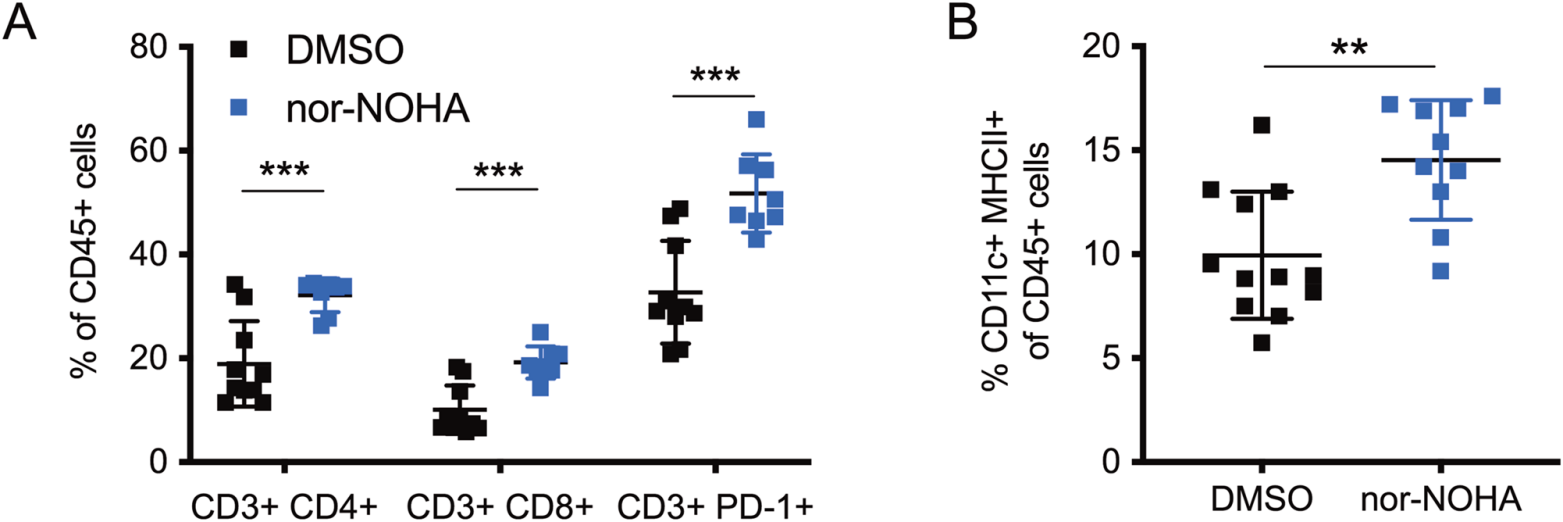
Transcutaneous arginase inhibition is associated with significantly more intratumoral T cells and dendritic cells in PDVC57 tumors. (**A**) Percentage of intratumoral CD45^+^ leukocytes that are CD3^+^ CD4^+^, CD3^+^ CD8^+^, or CD3^+^ PD-1^+^ indicating tumor-infiltrating T cells; CD3^+^ CD4^+^, CD3^+^ CD8^+^, CD3^+^ PD-1^+^ cells significantly higher in nor-NOHA-treated tumors. (**B**) Percentage of intratumoral CD45^+^ leukocytes that are CD11c^+^ MHCII^+^, indicating tumor-infiltrating dendritic cells; significantly higher in nor-NOHA-treated tumors. **P < 0.01; ***P < 0.001 using the unpaired *t*-test adjusted for multiple comparisons. Two separate experiments with n > 5 per group.

We demonstrated that direct cytotoxicity of nor-NOHA was not the mechanism of tumor growth suppression as an in vitro kill curve assay demonstrated a LD_50_ of 18.91 mM nor-NOHA in PDVC57 cells (Supplemental Fig. 4)—almost fourfold higher than the concentration used in the in vivo treatments. Further, as shown above, transcutaneously delivered nor-NOHA did not induce a significant reduction of tumor growth in vivo in *Rag1* KO mice (Fig. 5). Therefore, we sought to characterize the immunomodulatory effects of arginase inhibition. In B6 mice injected with PDVC57 tumors, nor-NOHA application increased CD4^+^ and CD8^+^ T cells, as well as CD11c^+^ MHCII^+^ dendritic cells within the tumor (Fig. 6A,B). Interestingly, proportions of CD3^+^ T cells expressing PD-1 also increased after treatment (Fig. 6A).

**Figure 7:**
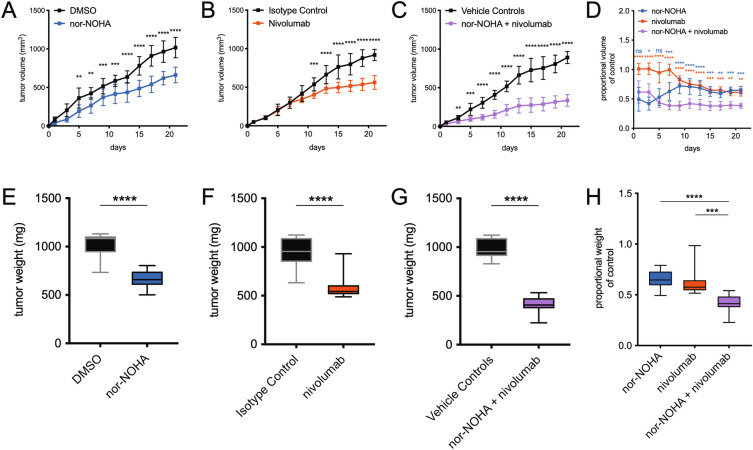
The combination of transcutaneous arginase inhibition and systemic PD-1 inhibition is synergistic in reducing PDVC57 tumor growth. (**A**) Growth curve of DMSO-treated compared to nor-NOHA-treated PDVC57 tumors, with a significant difference by day 5. (**B**) Growth curve of isotype control-treated compared to nivolumab-treated PDVC57 tumors, with a significant difference by day 11. (**C**) Growth curve of vehicle controls-treated compared to combination nor-NOHA + nivolumab-treated PDVC57 tumors, with a significant difference by day 3. (**D**) Proportional volume of nor-NOHA, nivolumab, and combination nor-NOHA + nivolumab compared to their respective vehicle controls; the combination treatment has significantly smaller proportional tumor volumes compared to either treatment alone. (**E**) Endpoint tumor weight of DMSO-treated compared to nor-NOHA-treated PDVC57 tumors; nor-NOHA-treated tumors were significantly lower in weight. (**F**) Endpoint tumor weight of isotype control-treated compared to nivolumab-treated PDVC57 tumors; nivolumab-treated tumors were significantly lower in weight. (**G**) Endpoint tumor weight of vehicle controls-treated compared to combination nor-NOHA + nivolumab-treated PDVC57 tumors; combination-treated tumors were significantly lower in weight. (**H**) Proportional endpoint tumor weight of nor-NOHA, nivolumab, and combination nor-NOHA + nivolumab treatments compared to their respective vehicle controls; the combination treatment has significantly smaller proportional tumor weights compared to either treatment alone. *P < 0.05; **P < 0.01; ***P < 0.001; ****P < 0.0001 using the unpaired *t*-test adjusted for multiple comparisons. Two separate experiments with n > 5 per group.

**Figure 8:**
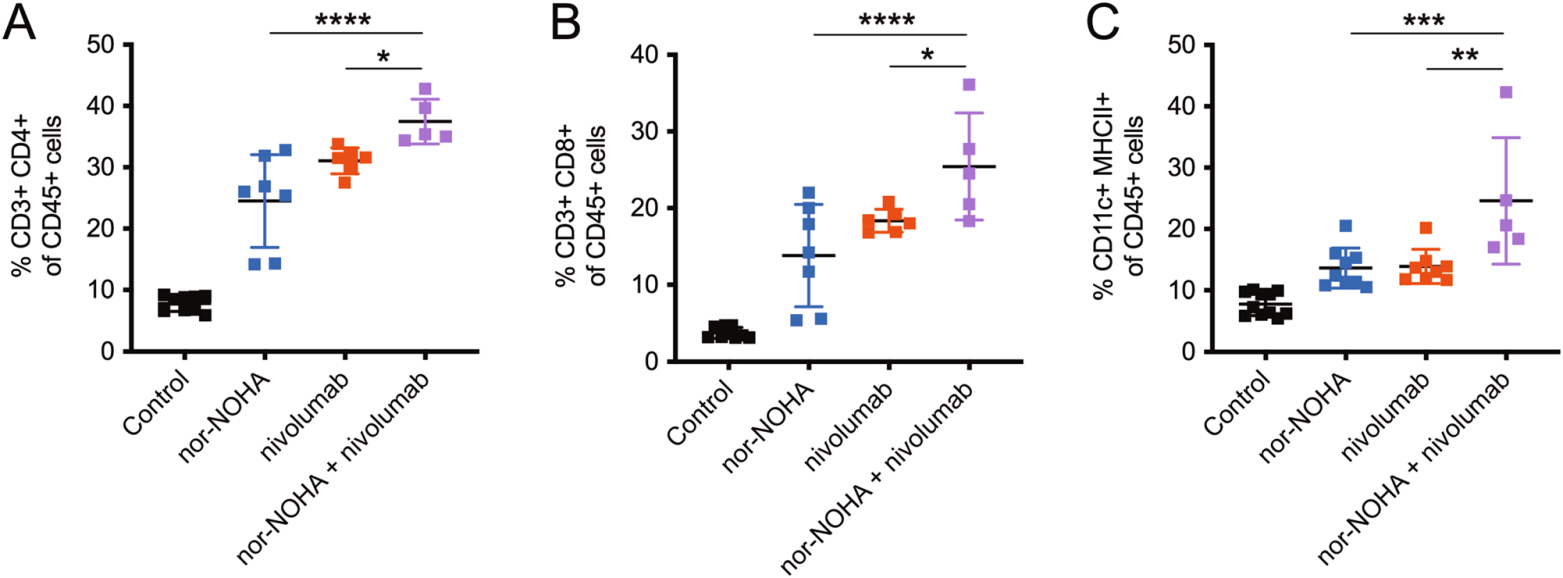
Combination treatment with transcutaneous arginase inhibition and systemic PD-1 inhibition is associated with increased recruitment of intratumoral T cells and dendritic cells than either treatment, alone. (**A**) Percentage of intratumoral CD45^+^ leukocytes that are CD3^+^ CD4^+^, indicating tumor-infiltrating CD4^+^ T cells; combination nor-NOHA + nivolumab treatments had significantly higher proportions than either treatment alone. (**B**) Percentage of intratumoral CD45^+^ leukocytes that are CD3^+^ CD8^+^, indicating tumor-infiltrating CD8^+^ T cells; combination nor-NOHA + nivolumab treatments had significantly higher proportions than either treatment alone. (**C**) Percentage of intratumoral CD45 + leukocytes that are CD11c^+^ MHCII^+^, indicating tumor-infiltrating dendritic cells; combination nor-NOHA + nivolumab treatments had significantly higher proportions than either treatment alone. *P < 0.05; **P < 0.01; ***P < 0.001; ****P < 0.0001 using the unpaired *t*-test adjusted for multiple comparisons. Two separate experiments with n > 5 per group.

Due to the increased PD-1 expression in nor-NOHA treated PDVC57 tumors, combination therapy with the PD-1 inhibitor nivolumab was tested. The combination therapy further decreased PDVC57 tumor growth (Fig. 7A–D) and final tumor weight (Fig. 7E–H), compared to either nor-NOHA or nivolumab alone. In addition, the combination treatment increased intratumoral CD4^+^ and CD8^+^ T cells, and CD11c^+^ MHCII^+^ dendritic cells in comparison to either nor-NOHA or nivolumab treatment, alone (Fig. 8A–C).

## Discussion

Immunotherapy is a rapidly emerging field in the treatment of cSCC and has largely focused on eliciting an adaptive immune response. The rationale behind treating cSCC by activating adaptive immunity is based on the observation that cSCC exhibits a high load of neoepitope mutations induced by ultraviolet (UV) radiation, which should stimulate cytotoxic T cells^30^. This mutational load was demonstrated in both PDV and PDVC57 cell lines through whole exome sequencing and prediction of more than 1900 neoepitope-contributing exonic variants (Supplementary Fig. 1). Cemiplimab is an anti-PD-1 checkpoint inhibitor recently approved in 2018 by the United States Food and Drug Administration for the treatment of metastatic and locally advanced cSCC^31–33^. Pembrolizumab, another PD-1 inhibitor, is currently being studied as another possible immunotherapeutic in cSCC^34–36^. However, only half of patients with unresectable locally advanced and/or metastatic cSCC responded to cemiplimab checkpoint inhibition^31^. As such, other targets in the immune response against cSCC may be exploited for further therapy.

TAM-derived arginase is increasingly becoming an important therapeutic target in various cancers. The degradation of arginine, in which arginase plays an integral part, has been demonstrated to lead to T cell suppression^37^. Clinically, the upregulation of arginase activity and the expression of *ARG1*, has been correlated with various cancers, such as gastric, breast, renal cell, and head and neck squamous cell carcinomas^38–41^. In this study, we sought to utilize transdermal arginase inhibition as a method of non-invasive drug delivery that is particularly applicable to cSCC, a skin cancer.

Testing this hypothesis required an appropriate murine model with an intact immune system. However, as noted previously, there is a lack of preclinical models available for testing of potential therapeutics, which drastically hinders the further development of new therapies. The current gold standard for analysis of cSCC in immunocompetent mice is chemical carcinogenesis using a two-stage protocol of 7,12-dimethylbenz[a]anthracene (DMBA) and 12-*O*-tetradecanoylphorbol-13-acetate (TPA) application^42,43^. This method is limited by the multiple-month delay between initiation of carcinogenesis and observation of clinically apparent tumors. Many of the tumors that develop are also more similar, in terms of histology and growth characteristics, to squamous papillomas than true cSCC^42–44^. Several injectable murine cSCC tumor models have been derived from DMBA or TPA treatment. HaCa4 were transformed by HaMSV-transforming retrovirus and TPA^45^. CarB and CarC were derived from two anaplastic skin carcinomas induced by DMBA and TPA^46^. The PDV cell line was derived from primary C57BL/6 mouse epidermal cells treated in vitro with DMBA^27^. None of these cell lines form tumors consistently in immunocompetent syngeneic mice^27,45,46^. Herein we characterized PDVC57, a cell line derived by in vivo passaging of the PDV cell line. Morphologically and genetically, we have shown that PDVC57 is a distinct cSCC cell line. Notably, both the PDV and PDVC57 cell lines bear the *Hras*^*Q61L*^ mutation, a well-characterized driver mutation in some human cSCC^47,48^. PDVC57 was able to consistently form cSCC tumors in immunocompetent B6 mice, and the tumorigenesis, as we have demonstrated, is facilitated in part by immunomodulation of the tumor microenvironment by the PDVC57 cells via increased arginase activity and decreases in intratumoral effector T cells and dendritic cells.

We demonstrated that transcutaneous arginase inhibition was sufficient to decrease growth of the PDVC57 tumors, in part due to an increase in intratumoral effector T cells and dendritic cells. The involvement of the adaptive immune system was further demonstrated given the lack of anti-tumorigenic effect when nor-NOHA was applied to *Rag1* KO mice, which lack mature T and B cells. Somewhat surprisingly, arginase inhibition in immunocompetent B6 mice also resulted in increased PD-1 expression on the intratumoral T cells. The mechanism of this is unclear; one possible pathway is that arginase inhibition leads to the increased activation of T cells with subsequent upregulation of the hyperactivation/exhaustion marker PD-1. The infiltration of PD-1^+^ T cells have been documented to be a favorable prognostic biomarker in both SCC of the head and neck and melanoma due to their response to checkpoint inhibition therapy^49,50^. As such, we were able to use systemic administration of the PD-1 inhibitor nivolumab in conjunction with topical nor-NOHA to decrease the rate of tumor growth more than either individual agent alone.

Finally, the development of cSCC due to UV radiation-induced carcinogenesis carries with it the relatively unique concept of field cancerization. Originally described in oropharyngeal SCC, field cancerization in cSCC is defined as the anatomical area adjacent to skin cancers and/or pre-cancers that demonstrate photodamage, and may show histopathologic dysplasia^51,52^. As such, treatment of only the pre-cancerous and/or cancerous lesions will not address the surrounding dysplastic tissue that carry similar mutational burdens and are likely to transform into pre-cancerous and/or cancerous lesions in the future^53^. Current field-directed therapies include 5-fluorouracil, a topical chemotherapeutic agent, and imiquimod, a toll-like receptor 7 agonist^54^. Topical arginase inhibition may provide an additional avenue of field-directed therapy for cSCC.

In summary, cSCC is the second-most commonly diagnosed cancer in the US and has significant morbidity and mortality rates, especially in high-risk populations. The use of immunotherapy such as checkpoint inhibition for cases of advanced cSCC has shown promise, though a majority of patients do not respond. In this study, we have characterized a pre-clinical injectable immunocompetent mouse model of cSCC and demonstrated the efficacy of topical arginase inhibition in reducing tumorigenesis of cSCC, especially in conjunction with checkpoint inhibitors. These results are promising for the development of future topical adjuvant therapies in the treatment of cSCC.

## Methods

### Study approval

The ethical use of the animals in this study was in accordance with policies set forth by the United States Department of Agriculture and the Public Health Service. This study was reviewed and approved by the Yale University Institutional Animal Care and Use Committee.

### Animals

C57BL/6J and B6.129S7-*Rag1*^*tm1Mom*^/J mice were purchased from The Jackson Laboratory. Seven- to ten-week-old female mice were used for all experiments.

### Cell lines

PDV cells were a gift of Michael Girardi, MD (Yale University, New Haven, CT). PDVC57 cells were obtained from Department of Cancer Biology, Instituto de Investigaciones Biomédicas Alberto Sols, Consejo Superior de Investigaciones Científicas (CSIC), Universidad Autónoma de Madrid (UAM), Madrid 28029, Spain.

### Cell culture and suspension

Cells were cultured in DMEM with pyruvate (GIBCO) and supplemented with 10% FBS (Gemini Bio-Products), 25 mM HEPES, 2 mM l-glutamine, and penicillin/streptomycin (GIBCO). Cell suspensions were made by incubating with trypsin–EDTA (GIBCO) for 10 min, neutralizing, and resuspending in appropriate volumes of PBS.

### PDVC57 injection

Mice were anesthetized with ketamine, flanks shaved, and using intradermal injection technique injected in the right flank with 50 μL of PDVC57 cells in suspension.

### Tumor measurements

Mice were sedated with isoflurane. Calipers were used to measure tumor diameter. Tumor volume was calculated using the following formula: (tumor length) × (tumor width)^2^ × 0.52, where length is longer than the width.

### Topical application of nor-NOHA

20–50 μL (depending on tumor size) of 5 mM nor-NOHA (EMD Millipore) in DMSO or DMSO control was applied twice daily, starting one day after injection of PDVC57 cells. Tumor sizes were measured as above every other day. At twenty-one days, the mice were euthanized using carbon dioxide and cervical dislocation. The tumors were then dissected out and weighed.

### Intravenous infusion of nivolumab

3 mg/kg nivolumab or isotype control was injected into the retro-orbital sinus of the mice once per week, beginning one day after injection of PDVC57 cells. Tumor sizes were measured as above every other day. At twenty-one days, the mice were euthanized using carbon dioxide and cervical dislocation. The tumors were then dissected out and weighed.

### Histology and immunofluorescence

Freshly collected tumors were either fixed in 4% paraformaldehyde and embedded in paraffin or placed in optimal cutting temperature compound (OCT). The prepared tumors were cut into 5 μm sections, with the paraffin-embedded sections used for hematoxylin and eosin (H&E) staining and the frozen sections for immunofluorescence. The H&E slides were visualized with light microscopy. The frozen section slides were stained with DAPI and anti-p63 antibody conjugated to FITC (Bioss, bs-0723R-FITC) and scanned with Leica SP5 confocal microscope.

### Whole exome sequencing

Exome sequencing and mutational burden analysis were performed according to^55^. Neoantigen prediction was performed for nonsynonymous SNV, Frameshift, Indel, and Stop-Loss variants that passed MuTect2 quality control filters to create all possible 8mers, 9mers, 10mers, and 11mers using custom Python scripts. Peptide binding affinity of wildtype and mutant peptides to C57BL6/J MHC I alleles H2-Db and H2-Kb were calculated using netMHCCons^56^.

### Tumor processing

In a sterile Petri dish (non-tissue-culture-treated plate), a digestion buffer of 20 mL PBS + Ca^2+^ + 200μL Liberase (5 mg/mL, Roche) + 1 mL DNAse (2 mg/mL, Roche) was prepared. After adding the tumor to the Petri dish, the tumor was minced with scissors and then more finely with a razor blade. The tumor was then transferred into a 50 mL tube and incubated with shaking for 30 min at 37 °C. The digested tumor was then placed on ice for 3 min with the addition 200μL of 0.5 M EDTA (GIBCO). The tumor suspension was filtered through a 70 μM cell strainer (Corning, Falcon) into another 50 mL tube. The original tube was washed once with 10 mL of PBS and again filtered through the strainer. The filtered tumor suspension was then centrifuged for 5 min at 1300 RPM at 4 °C. The supernatant was discarded. Between 2 and 10 mL of ACK lysis buffer (GIBCO) was added to lyse red blood cells. After 5 min, the lysis reaction was stopped with approximately 10 mL of PBS + 0.5% FBS (FACS buffer). The resulting suspension was filtered through a 70 μM cell strainer into another 50 mL tube. The filtered contents were centrifuged for 5 min at 1300 RPM at 4 °C. Supernatant was discarded.

### Staining for flow cytometry

Fc-block was added to dissociated tumor cell suspensions at 1:200 dilution and incubated for 20 min on ice. Three panels of flow cytometry antibodies were used for leukocyte staining—Panel 1: CD11b-ef450 (M1/70) #48-0112-82 Invitrogen, MHCII-FITC (M5/114.15.2) #11-5321-81, F4/80-AF700 (BM8) #56-4801-82, CD11c-PECy7 (N418) #25-0114-82 eBioscience, CD45-APC (30-F11) #10311 and Live/Dead-AmCyan (#423101) Biolegend; Panel 2: CD45-PerCP-Cy5.5 (30-F11) #103132, CD3e-APC (145-2C11) #100312, B220 BV605 (RA3-6B2) #103243, PD-1-PE (29F.1A12) #135205, CD4-BV421 (GK1.5) #100437, CD8a-APC Fire 750 (53-6.7) #100766 and Live/Dead-AmCyan (#423101) Biolegend; Panel 3: CD206-FITC (MR5D3) #MA5-16870, CD11b-ef450 (M1/70) #48-0112-82 Invitrogen, F4/80-AF700 (BM8) #56-4801-82 eBioscience, intracellular Arg1-APC (#IC5868A) R&D systems, MHCII-APC-Cy7 (M5/114.15.2) #107652, CD45-PerCP-Cy5.5 (30-F11) #103132 and Live/Dead-AmCyan (#423101) Biolegend.

### Flow cytometry analysis

Approximately 200–500 μL FACS buffer were added to the tumor cell pellets in order to resuspend them. Greater volumes were added with a higher number of cells. Cells were analyzed on a LSRII (BD Biosciences).

### Arginase activity assay

Arginase activity was detected using the Arginase Activity Colorimetric Assay Kit (BioVision, Milpitas, CA). Tumors were processed as above and lysed in assay buffer at a concentration of 1 × 10^6^ cells/100 µL. The assay was conducted in accordance with manufacturer instructions. The tumor lysates and background samples were measured in triplicate in a 96 well plate. (Corning, Falcon) The plate was warmed to 37 °C and read at 570 nm (BioTek). Optical density was recorded in kinetic mode every 5 min, beginning at time zero until 60 min, or signal saturation. Arginase Activity (Units [U]) was calculated by fitting the corrected sample reading to the generated standard curve.

### Statistics

Some data and graphs were created using Prism 7 (GraphPad Software). Statistical tests were performed as described in the figure legends, and when possible, adjusted *P* values were used to assess for significance, with a threshold of adjusted *P* < 0.05.

This study was carried out in compliance with the ARRIVE 2.0 guidelines.

## Supplementary Information

**Supplementary Figures.**
