# Supplementary material for: Topical arginase inhibition decreases growth of cutaneous squamous cell carcinoma
Source: Sci Rep. 2021 May 24;11:10731. doi: 10.1038/s41598-021-90200-y (PMC8144401; doi:10.1038/s41598-021-90200-y)

Supplemental Figures

Topical Arginase Inhibition Decreases Growth of Cutaneous Squamous Cell Carcinoma

Amit Mittal, MD[1,*], Mike Wang, MD, MHS[1,2,*,†], Aurobind Vidyarthi, PhD[1], Diana Yanez, BS[1], Gabriela Pizzurro, PhD[1], Durga Thakral, MD, PhD[1], Erin Tracy, MS[3], Oscar R. Colegio, MD, PhD[3,4,†]

[1]Department of Dermatology, Yale School of Medicine,
[2]Department of Medicine, Massachusetts General Hospital
Departments of [3]Immunology and [4]Dermatology, Roswell Park Comprehensive Cancer Center
**indicates equal contributions*
[†]indicates corresponding authors

Mike Wang, MD, MHS
Department of Medicine
Massachusetts General Hospital
55 Fruit Street
Boston, Massachusetts 02114

203-809-9089: Cell
617-726-2000: Office

mwang43@mgh.harvard.edu

Oscar Colegio, MD, PhD
Department of Dermatology
Roswell Park Comprehensive Cancer Center
Elm and Carlton Streets
Buffalo, New York 14263

203-640-9504: Cell
716-845-3056: Fax
716-845-8598: Office

oscar.colegio@roswellpark.org

Conflict of interest statement

“The authors have declared that no conflict of interest exists.”

Comparison		PDVC57 v. Blood	PDV v. Blood	PDV v. PDVC57	PDVC57 v. PDV
**All Variants**		6657	6910	730	390
**Total # Exonic Mutations**		2726	2896	311	176
**Exonic Variant Type**	Synonymous SNV	620	657	68	40
	Nonsynonymous SNV	1901	2019	221	129
	Frameshift deletion	25	22	4	3
	Frameshift insertion	13	18	5	2
	Nonframeshift deletion	2	1	0	1
	Nonframeshift insertion	0	0	0	0
	Stoploss	4	4	0	0
	Stopgain	144	157	13	1
	NA	0	0	0	0
	Unknown	17	18	0	0
**Number of Neoepitope-contributing Variants (Nonsynonymous SNV, Frameshift, Indel, and Stop-Loss)**		1945	2064	230	135

Supplemental Figure 1. Summary of variant types in each tumor versus normal blood comparison. PDV and PDVC57 demonstrate similar numbers of neoepitope-contributing variants.

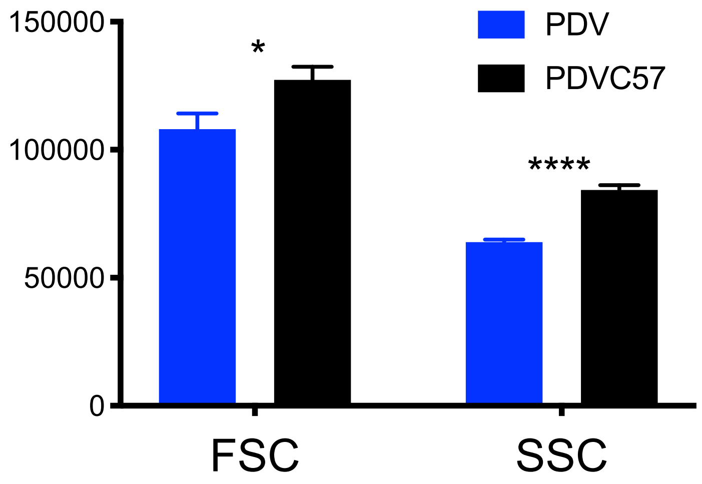

Supplemental Figure 2. PDVC57 cells were significantly larger, as shown by forward scatter (FSC), and significantly more granular, as shown by side scatter (SSC), than PDV cells. *P < 0.05, ****P < 0.0001 by Student’s t test; n = 3 per group.

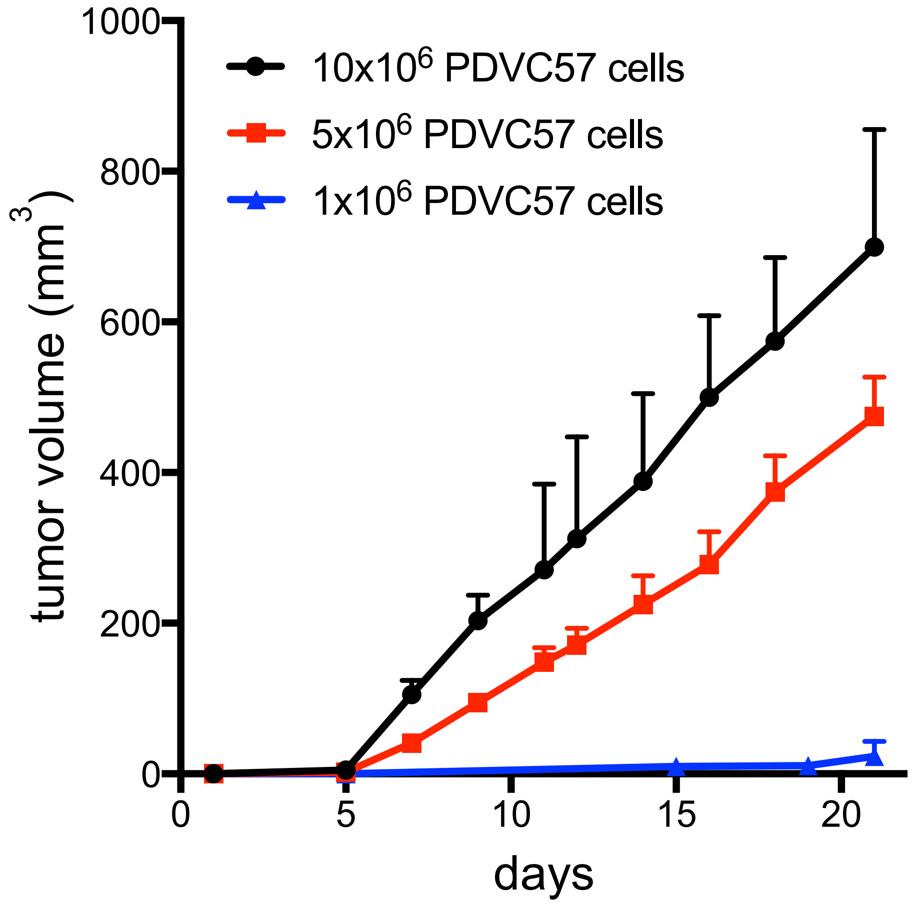

Supplemental Figure 3. All (5/5) mice injected with 5 and 10 million PDVC57 cells developed tumors, while only 60% (3/5) of the mice injected with 1 million PDVC57 cells developed tumors.

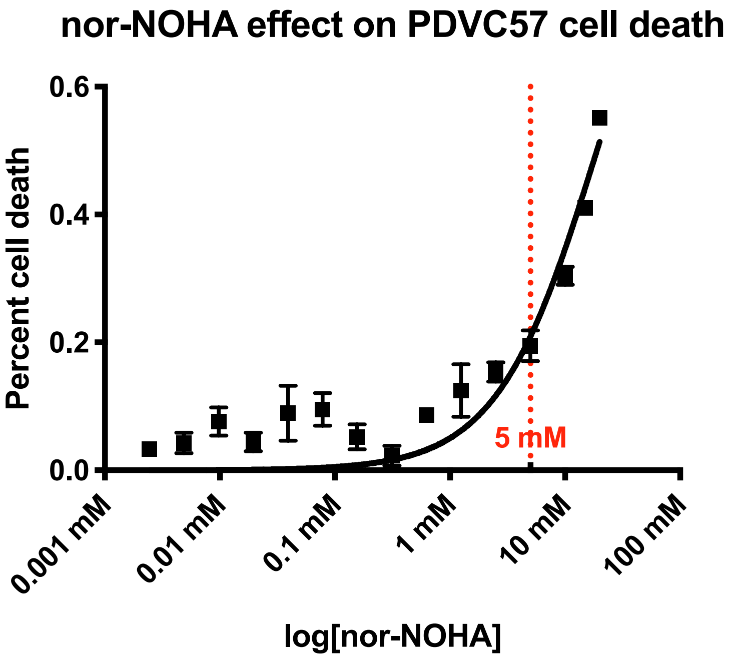

Supplemental Figure 4. *In vitro* kill curve assay of nor-NOHA on PDVC57 demonstrates a LD_{50} of 18.91 mM, while the concentration used in *in vivo* experiments (5 mM) is indicated by the red dotted line.

Supplemental Figure 5. Representative flow cytometry plots of Main Figure 4.

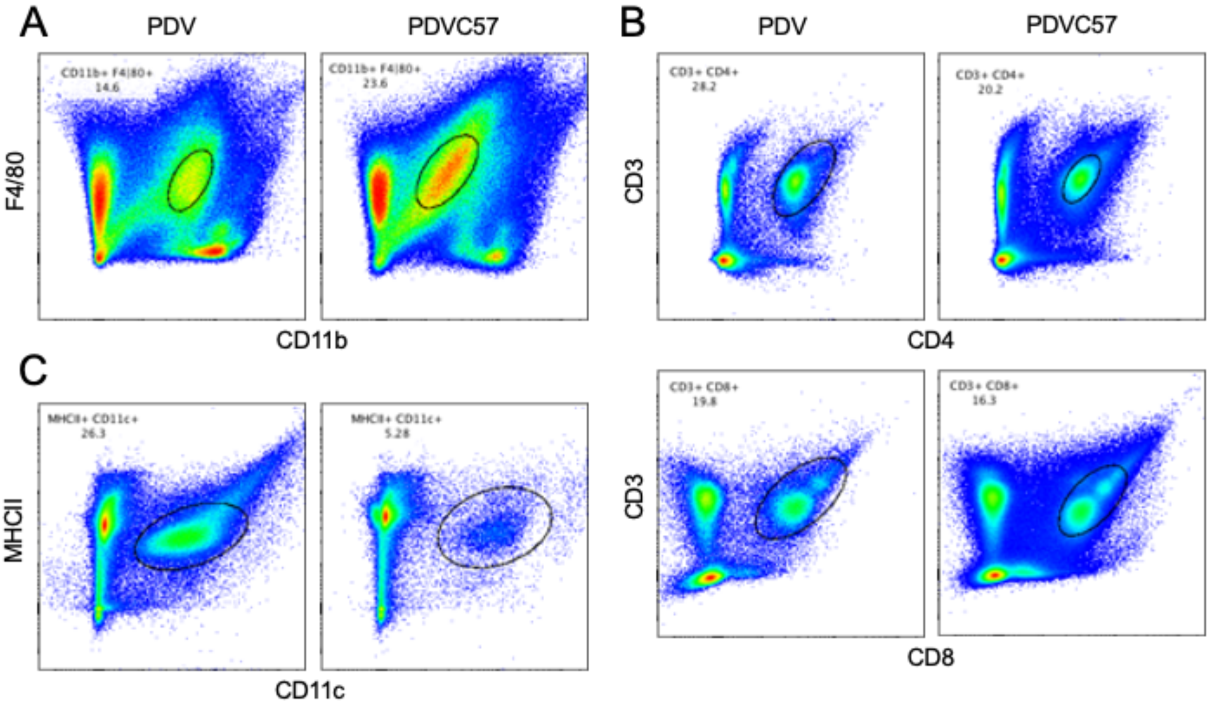

Supplemental Figure 6. Representative flow cytometry plots of Main Figure 6.

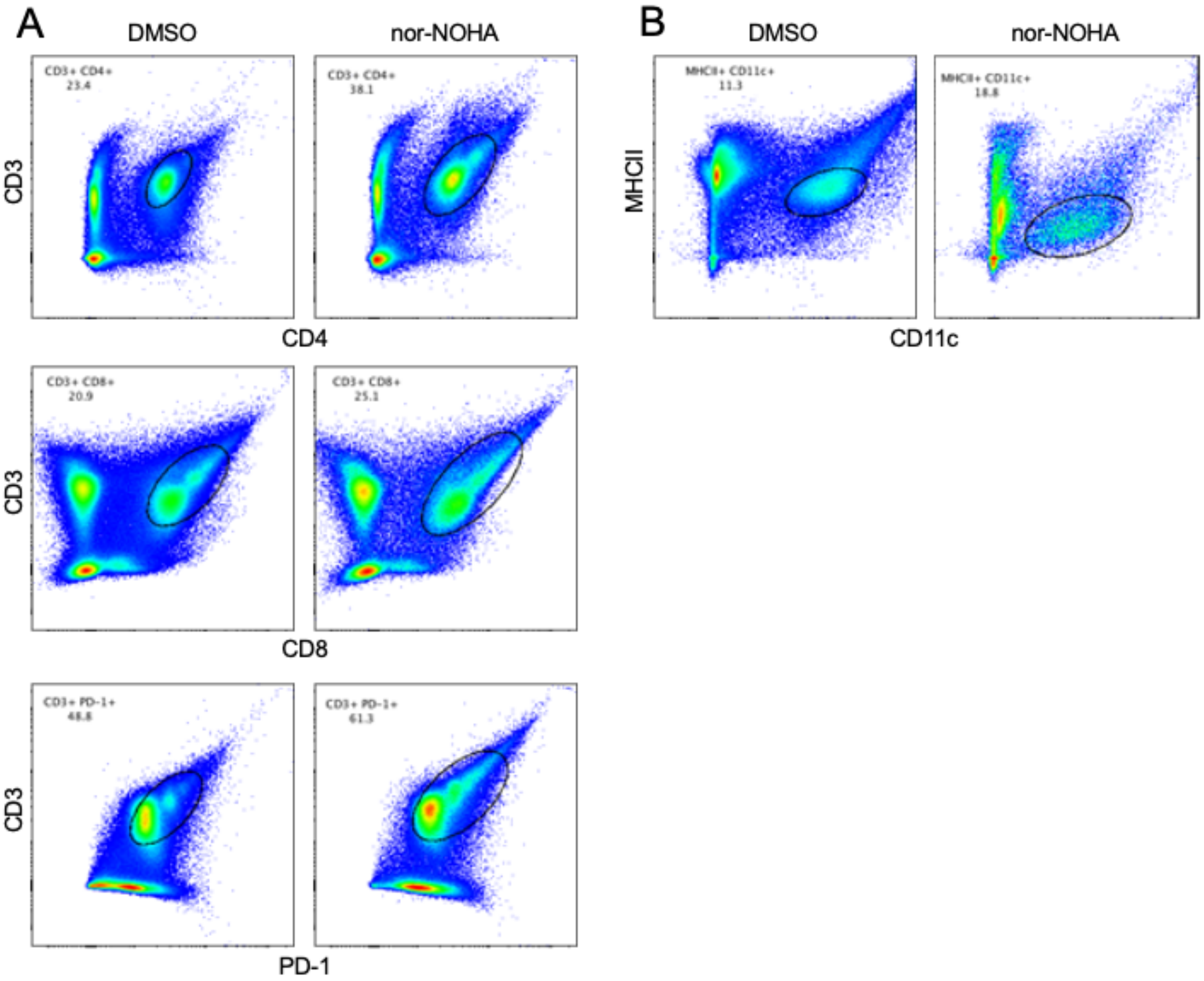

Supplemental Figure 7. Representative flow cytometry plots of Main Figure 8.

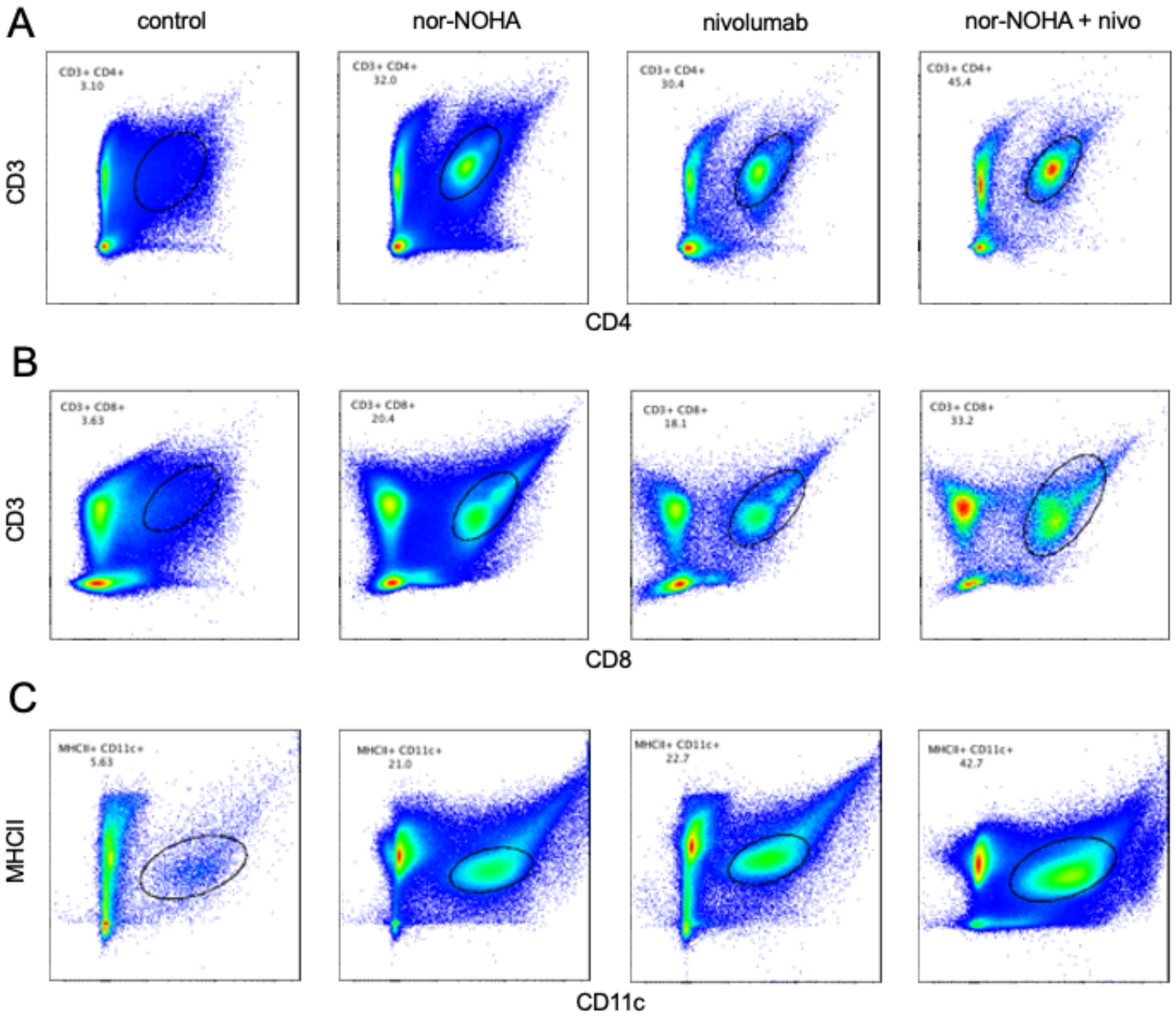

Supplement: Supplementary file 1 — Supplementary Figures. [file 41598_2021_90200_MOESM1_ESM.pdf]
